# The Impact of Lymphovascular Space Invasion on Recurrence and Survival in FIGO Stage I Node-Negative Endometrioid Endometrial Cancer

**DOI:** 10.3390/jcm14186535

**Published:** 2025-09-17

**Authors:** Yakup Yalcin, Bahadir Kosan, Serenat Yalcin, Merve Abay, Kemal Ozerkan

**Affiliations:** 1 Department of Obstetrics and Gynecology, School of Medicine, Bursa Uludag University, Bursa 16110, Turkey; 2 Department of Obstetrics and Gynecology, Ali Osman Sonmez Oncology Hospital, Bursa 16110, Turkey

**Keywords:** endometrial cancer, early stage, lymphovascular space invasion, disease-free survival, overall survival, recurrence

## Abstract

**Background/Objective**: To evaluate the prognostic impact of lymphovascular space invasion (LVSI) on disease-free survival (DFS) and overall survival (OS) in patients with FIGO 2009 stage I endometrioid endometrial cancer with pathologically negative lymph node involvement. **Methods**: This retrospective cohort study included 469 patients with FIGO 2009 stage I node-negative endometrioid endometrial carcinoma who underwent comprehensive surgical staging at a single tertiary center between January 1993 and April 2025. Demographic, clinicopathological, treatment, and follow-up data were collected. Survival outcomes were assessed using Kaplan–Meier analysis, and prognostic factors were identified via univariate and multivariate Cox regression models. **Results**: LVSI was present in 17.7% of the cohort (*n* = 83). Patients with LVSI had significantly higher tumor grades, larger tumor size, and deeper myometrial invasion compared to LVSI-negative patients (*p* < 0.001). Recurrence was more frequent in the LVSI-positive group (14.5% vs. 6.5%, *p* = 0.026), with distant metastasis predominating (83.3%). The 5-year DFS was 86.4% in the LVSI-positive group versus 96.3% in the LVSI-negative group (*p* = 0.0020), while the 5-year OS was 72.1% vs. 91.2%, respectively (*p* = 0.0014). In multivariate analysis, LVSI was an independent prognostic factor for both recurrence (HR = 4.80, 95% CI: 1.62–14.21; *p* < 0.001) and overall mortality (HR = 3.33, 95% CI: 1.43–7.77; *p* = 0.012). **Conclusions**: LVSI is a strong and independent predictor of adverse oncologic outcomes in early-stage, node-negative endometrioid endometrial cancer. Its presence is associated with significantly decreased DFS and OS, particularly due to an increased risk of distant recurrence. These findings support the incorporation of LVSI into contemporary risk stratification and adjuvant treatment algorithms.

## 1. Introduction

Endometrioid endometrial cancer (EEC) is the most common histologic subtype of endometrial malignancy and is generally associated with a good clinical prognosis when diagnosed at an early stage. However, recurrence is reported in approximately 2% to 10% of International Federation of Obstetrics and Gynecology (FIGO) 2009 stage I patients [1].

The Cancer Genome Atlas (TCGA) project contributed to defining the molecular classification of endometrial cancer. Following the release of TCGA’s findings, multiple studies have identified four subgroups—POLE-ultramutated (POLEmut), mismatch repair-deficient (MMRd), p53-abnormal (p53abn), and no specific molecular subgroup (NSMP)—using surrogate markers that parallel the original classification [2].

The 2021 ESGO/ESTRO/ESP guidelines incorporated both known and unknown molecular classifications into their risk stratification system, taking into account pathological factors such as LVSI, tumor stage, and histologic grade [3]. In contrast, the updated 2025 ESGO/ESTRO/ESP guidelines have adopted an entirely molecular-based classification system. In this revised model, the four molecular subtypes—POLE-ultramutated (POLEmut), mismatch repair-deficient (MMRd), p53-abnormal (p53abn), and no specific molecular profile (NSMP)—are integrated with disease stage and additional biological characteristics to define low, intermediate, high-intermediate, high, and uncertain risk categories [4]. Each risk group is now associated with specific adjuvant treatment recommendations, tailored according to the molecular profile and complementary prognostic factors.

In women with early-stage endometrial cancer, poor prognostic factors such as deep myometrial invasion, high histological grade, and the presence of lymphovascular space invasion (LVSI) have been identified by various researchers [5,6,7]. Lymphovascular space invasion is a pathological condition characterized by the presence of tumor cells within lymphatic channels or small blood vessels located outside the primary tumor mass. It has been reported in up to 35% of all endometrial cancer cases and in approximately 10–15% of patients with FIGO stage I disease [8]. LVSI is frequently associated with other uterine risk factors, such as high tumor grade and deep myometrial invasion. Numerous studies have established LVSI as an independent prognostic indicator of poor outcomes in patients with FIGO stage I EEC. Specifically, LVSI has been linked to reduced disease-free survival (DFS) and overall survival (OS), as well as an increased risk of distant recurrence and lymph node (LN) metastasis. Notably, its presence has also been associated with poorer survival outcomes in node-negative patients, suggesting a possible contribution of hematogenous dissemination [9,10,11,12]. LVSI is considered one of the strongest predictors of recurrence in patients with FIGO 2009 stage I endometrioid endometrial cancer; therefore, it is of particular importance in adjuvant treatment planning [13].

Given the prognostic significance of LVSI in the literature, the NCCN, GOG-99, and ESGO guidelines use LVSI as a criterion for classifying endometrial cancer patients into low-, intermediate-, and high-risk groups. Furthermore, the extent of lymphovascular space invasion has been included as a staging criterion in the 2023 FIGO endometrial cancer staging system. In this context, the current definition of substantial LVSI, as proposed by the World Health Organization (WHO), is used [3,4,14,15].

The aim of this study was to evaluate the prognostic significance of lymphovascular space invasion on disease-free survival and overall survival in women with FIGO 2009 stage I endometrioid endometrial cancer without lymph node involvement who underwent surgical lymph node evaluation.

## 2. Materials and Methods

This retrospective cohort study was conducted using clinical and histopathological data obtained from the electronic medical records of patients diagnosed with endometrial cancer and treated or followed at Bursa Uludağ University Hospital between January 1993 and April 2025. The study received ethical approval from the Bursa Uludag University Clinical Research Ethics Committee (Protocol No. 2021-10/26) and was conducted in accordance with the Declaration of Helsinki.

### 2.1. Patient Selection

Patients who underwent surgical staging for endometrial cancer, including total hysterectomy, bilateral salpingo-oophorectomy, pelvic lymphadenectomy, and/or para-aortic lymphadenectomy via laparotomy or laparoscopy, were considered for inclusion. According to institutional practice, para-aortic lymphadenectomy was not routinely performed in low-risk patients with tumors smaller than 2 cm, less than half myometrial invasion, and grade 1–2 tumors.

Inclusion criteria were 1. histologically confirmed endometrioid endometrial carcinoma, 2. surgically staged FIGO 2009 stage I disease, and 3. absence of lymph node metastasis on pathologic examination. Exclusion criteria comprised non-endometrioid histologic subtypes, lymph node involvement, unknown LVSI status, mixed histology, synchronous malignancies, lack of lymph node assessment during surgery, and receipt of neoadjuvant therapy. Due to the lack of sufficient infrastructure for sentinel lymph node in our center, a low number of patients were studied and these patients were excluded from the study.

### 2.2. Data Collection and Pathological Assessment

Demographic and clinical variables recorded included age, body mass index (BMI), menopausal status, tumor size, tumor grade, depth of myometrial invasion, lymphovascular space invasion, and use of adjuvant therapy. LVSI was defined histologically as the presence of viable tumor cells within endothelial-lined lymphatic or vascular spaces, located outside the main tumor mass. Evaluation was performed using hematoxylin and eosin–stained slides, with emphasis on cohesive tumor clusters at the invasive front that conformed to vascular architecture. Tumor size was defined as the largest diameter reported in the pathology records. Myometrial invasion was categorized based on the depth of infiltration. Patients were stratified into three risk groups based on stage, histologic grade, and LVSI status. The low-risk group included patients with stage IA, grade 1 or 2 tumors, and negative LVSI. The intermediate-risk group consisted of patients with stage IB, grade 1 or 2 tumors with negative LVSI, or stage IA, grade 3 tumors with negative LVSI. The high–intermediate-risk group included patients with stage IA or IB tumors with positive LVSI, and stage IB, grade 3 tumors regardless of LVSI status.

### 2.3. Follow-Up and Outcomes

All pathology reports were discussed at a multidisciplinary tumor board, and decisions regarding adjuvant treatment were made accordingly. Postoperative follow-up was conducted every 3–6 months during the first 2–3 years and every 6 months thereafter up to 5 years. Follow-up of patients who completed their 5th year continued annually. Imaging studies were ordered based on clinical symptoms or suspicion of recurrence. Recurrences were classified as locoregional (vaginal, pelvic lymph nodes, bladder, rectum), distant (abdominal cavity, para-aortic lymph nodes, lung, liver, bone, brain), or both if present simultaneously.

The primary endpoint was disease-free survival, defined as the interval from surgery to the first recurrence, death due to disease, or last follow-up. The secondary endpoint was overall survival, defined as the interval from surgery to death from any cause or last known survival. Deaths were classified into two groups: cancer-related or non-cancer-related. Recurrence and survival data were collected through clinical visits and institutional electronic databases.

### 2.4. Statistical Analysis

All statistical analyses were conducted using IBM SPSS Statistics for Windows, version 26.0 (IBM Corp., Armonk, NY, USA). Continuous variables were assessed for normality using the Shapiro–Wilk test. As the majority of the variables did not exhibit a normal distribution, non-parametric methods were applied. Specifically, Mann–Whitney U tests were used for comparison of continuous variables between groups, while Pearson’s Chi-square test or Fisher’s exact test was employed for categorical variables, depending on cell counts. Descriptive statistics were presented as median (range) for continuous variables and number (percentage) for categorical variables. A two-sided *p*-value < 0.05 was considered statistically significant in all analyses.

Survival outcomes, including disease-free survival and overall survival, were estimated using the Kaplan–Meier method, and differences between survival curves were evaluated using the log-rank test. DFS was defined as the time (in months) from the date of primary surgical treatment to the date of the first documented recurrence or the date of last follow-up without recurrence. OS was defined as the time from surgery to the date of death from any cause or the last known follow-up. To identify independent prognostic factors affecting DFS and OS, Cox proportional hazards regression analyses were performed. Initially, univariate Cox regression was applied to assess the impact of each clinical and pathological parameter on survival outcomes. Variables with a *p*-value less than 0.10 in univariate analysis were subsequently included in the multivariate Cox regression models. Results were reported as hazard ratios (HRs) with corresponding 95% confidence intervals (CIs).

## 3. Results

**Figure 1:**
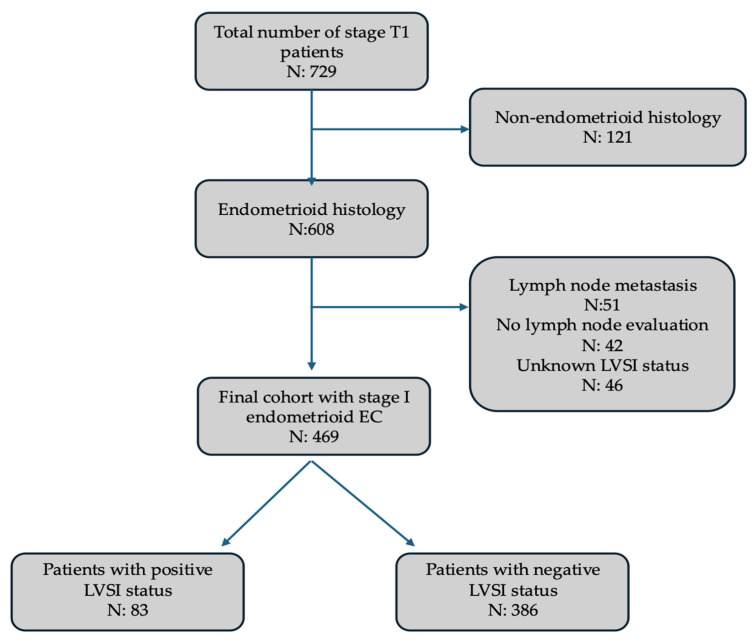
Patient selection flowchart for the study cohort of Stage I endometrioid endometrial cancer.

A total of 729 patients diagnosed with FIGO stage T1 endometrial cancer were evaluated. After applying strict eligibility criteria, 260 patients were excluded from the analysis for the following reasons: non-endometrioid histologic subtype (*n* = 121), presence of lymph node metastasis (*n* = 51), absence of lymph node evaluation at the time of surgery (*n* = 42), or unknown lymphovascular space invasion status (*n* = 46). As a result, the final study population included 469 patients with histologically confirmed endometrioid endometrial carcinoma, surgically staged as FIGO 2009 stage I, and pathologically confirmed to have no lymph node involvement. Among these, LVSI was identified in 17.7% of cases (*n* = 83), while the remaining 82.3% of patients (*n* = 386) demonstrated no evidence of LVSI on final pathology (Figure 1).

Table 1 summarizes patient characteristics. The median age was 61.4 years (range: 27–90) and the median BMI was 34.7 kg/m^2^ (range: 18.0–65.35); 91.9% were postmenopausal, with no significant group differences (*p* > 0.05). The median number of para-aortic and pelvic lymph nodes removed was 10.8 (range: 1–46) and 19.1 (range: 2–82), respectively, also without significant differences. Surgical approach distribution was similar between groups (laparoscopy vs. laparotomy, *p* = 0.925). LVSI-positive patients had significantly larger tumors (median 4.5 cm vs. 3.3 cm, *p* < 0.001) and higher tumor grades (grade 3: 20.5% vs. 12.4%, *p* < 0.001). Deep myometrial invasion (≥50%) was more frequent in LVSI-positive patients (49.4% vs. 22.3%, *p* < 0.001). Stage IB disease was also more common in the LVSI-positive group (48.2% vs. 22.8%, *p* < 0.001). Adjuvant therapy patterns differed significantly (*p* < 0.001): LVSI-positive patients more frequently received EBRT (66.3% vs. 38.1%) and chemotherapy (9.6% vs. 2.6%), while LVSI-negative patients more often had no adjuvant treatment (43.0% vs. 3.6%). Median follow-up for survivors was 58.1 months. Recurrence was higher in LVSI-positive patients (14.5% vs. 6.5%, *p* = 0.026), with distant recurrences predominating (83.3% vs. 48.0%, *p* = 0.037). Overall mortality was 34.9% in the LVSI-positive group vs. 25.1% in LVSI-negative patients (*p* = 0.091), with no significant difference in cancer-related death proportion (*p* = 0.495). Analysis of LVSI distribution across risk groups revealed a strong association between LVSI status and risk classification (*p* < 0.001). LVSI positivity was concentrated entirely in the high–intermediate risk group (83 patients, 100%), indicating that LVSI is a distinctive pathological feature particularly characteristic of this group.

**Figure 2:**
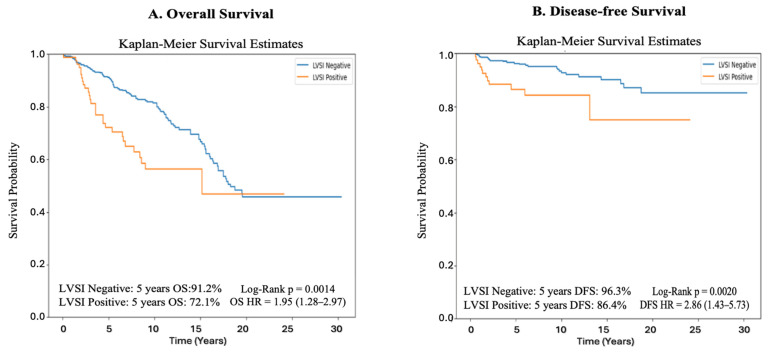
Kaplan-Meier curve for disease-free survival and overall survival in patients with pathologic stage T1N0 endometrial cancer, stratified by the presence or absence of lymphovascular space invasion (LVSI).

The 5-year overall survival rate was 91.2% in LVSI-negative patients compared to 72.1% in those with LVSI. This difference was statistically significant (Log-rank *p* = 0.0014). According to univariate Cox regression analysis, the presence of LVSI nearly doubled the risk of overall mortality (HR = 1.95; 95% CI: 1.28–2.97). Similarly, the 5-year disease-free survival rate was 96.3% in LVSI-negative patients and 86.4% in LVSI-positive patients. This difference was also statistically significant (Log-rank *p* = 0.0020). Univariate Cox analysis demonstrated that LVSI was associated with a nearly threefold increased risk of disease recurrence (HR = 2.86; 95% CI: 1.43–5.73) (Figure 2).

The variables included in the multivariate Cox regression model were selected based on two main criteria: (1) established prognostic relevance in FIGO stage I endometrioid endometrial cancer as documented in the literature, and (2) statistical or potential clinical significance in the univariate analysis of our cohort. Specifically, LVSI positivity, the primary variable of interest, has been consistently identified as a strong independent predictor of poor outcomes. Age > 60 years, BMI, and menopausal status were included due to their recognized influence on tumor biology and survival. High histologic grade (grade 3), tumor size ≥ 2 cm, and deep myometrial invasion (>50%) were selected given their well-documented associations with recurrence and lymphatic spread. Para-aortic lymph node dissection, surgical approach (laparotomy), and FIGO 2009 stage IB were incorporated to evaluate the potential impact of surgical factors and disease stage on survival. Adjuvant treatment status was included to account for treatment-related effects. Finally, recurrence occurrence and recurrence site (distant) were entered into the model to assess their prognostic impact on overall survival [3,4,14,16].

### 3.1. Univariate Analysis and Multivariate Analysis of Overall Survival

In the univariate Cox regression analysis, the presence of lymphovascular space invasion (LVSI) was significantly associated with decreased overall survival (OS) (HR: 3.91; 95% CI: 1.92–8.01; *p* < 0.001). Similarly, age > 60 years (HR: 1.83; 95% CI: 1.23–2.62; *p* = 0.005), grade 3 tumor (HR: 2.51; 95% CI: 1.13–5.57; *p* = 0.041), tumor diameter ≥ 2 cm (HR: 2.03; 95% CI: 1.12–3.74; *p* = 0.023), and FIGO stage IB (HR: 2.24; 95% CI: 1.14–5.12; *p* = 0.049) were also significantly associated with worse OS. Although not statistically significant, menopause status (*p* = 0.084), MI > 50% (*p* = 0.094), and para-aortic lymphadenectomy (*p* = 0.133) showed a trend toward poorer survival. The presence of recurrence (HR: 5.73; 95% CI: 2.91–11.33; *p* < 0.001) and distant recurrence site (HR: 3.29; 95% CI: 1.55–6.82; *p* = 0.002) were also strongly associated with reduced OS. In the multivariate Cox model, LVSI remained an independent prognostic factor for worse OS (HR: 3.33; 95% CI: 1.43–7.77; *p* = 0.012). Adjuvant treatment was also independently associated with decreased survival (HR: 5.45; 95% CI: 1.02–29.10; *p* = 0.047). Recurrence (HR: 4.84; 95% CI: 1.94–11.84; *p* < 0.001) and distant recurrence (HR: 2.52; 95% CI: 1.12–5.73; *p* = 0.032) remained significant predictors of poorer OS. Other factors such as age, tumor grade, tumor diameter, surgical approach, FIGO stage, and para-aortic lymph node dissection lost statistical significance in the multivariate model (Table 2).

### 3.2. Univariate Analysis and Multivariate Analysis of Disease-Free Survival

In univariate Cox regression analysis, the presence of lymphovascular space invasion (LVSI) was significantly associated with worse DFS (HR: 4.21; 95% CI: 2.02–8.61; *p* < 0.001). Other factors that significantly decreased DFS included grade 3 tumors (HR: 2.05; 95% CI: 1.06–4.35; *p* = 0.031), tumor size ≥ 2 cm (HR: 2.23; 95% CI: 1.14–4.49; *p* = 0.037), deep myometrial invasion (HR: 2.37; 95% CI: 1.01–5.38; *p* = 0.045), and FIGO 2009 stage IB (HR: 2.17; 95% CI: 1.08–4.35; *p* = 0.041). Although not statistically significant, postmenopausal status, para-aortic lymphadenectomy, and receipt of adjuvant treatment showed trends toward reduced DFS. The presence of recurrence (HR: 6.21; 95% CI: 3.82–10.71; *p* < 0.001) and distant recurrence site (HR: 2.45; 95% CI: 1.12–4.65; *p* < 0.001) were also strongly associated with reduced DFS. In the multivariate Cox regression model, LVSI remained an independent predictor of reduced DFS (HR: 4.80; 95% CI: 1.62–14.21; *p* < 0.001). Additionally, deep myometrial invasion (HR: 2.82; 95% CI: 1.24–2.93; *p* = 0.023), receipt of adjuvant treatment (HR: 4.32; 95% CI: 1.02–26.13; *p* = 0.044), recurrence (HR: 6.12; 95% CI: 4.93–9.77; *p* < 0.001), and distant recurrence site (HR: 1.53; 95% CI: 1.34–6.54; *p* < 0.001) were also found to be independently associated with worse DFS. Variables such as age, BMI, menopausal status, tumor size, surgical approach, and FIGO stage were not significant in the multivariate model (Table 3).

## 4. Discussion

In this retrospective cohort study, we evaluated 469 patients with FIGO 2009 stage I endometrioid endometrial cancer who were surgically staged and had no pathological lymph node involvement. The primary aim of this study was to investigate the prognostic significance of lymphovascular space invasion and its potential influence on clinical management. Our findings demonstrate that the presence of LVSI is significantly and independently associated with worse pathological features, increased recurrence rates, and reduced survival outcomes.

The evaluation of LVSI in endometrial cancer has traditionally been based on qualitative classification, with findings categorized as “present,” “suspicious,” or “absent.” However, increasing attention has recently been directed toward semiquantitative evaluation methods, particularly following the pooled analyses of the PORTEC-1 and PORTEC-2 trials. These analyses demonstrated a significant association between substantial LVSI—characterized by diffuse or multifocal involvement—and both higher rates of pelvic nodal recurrence and decreased overall survival [9,12,16,17,18]. In an effort to refine risk stratification, subsequent investigations sought to determine an optimal threshold for the number of involved vessels correlating with adverse outcomes. Nonetheless, the lack of routine lymphadenectomy in the PORTEC studies has led to the hypothesis that the prognostic implications of LVSI might, at least in part, be attributable to occult nodal metastases. Supporting this notion, a recent study by Pifer et al., which included only node-negative patients, reported no significant correlation between the extent of LVSI and oncologic outcomes; however, it is noteworthy that the study applied the threshold for substantial LVSI was ≥4 vessels [18]. However, the study by Peters et al. also demonstrated no significant difference in prognosis when the threshold was 2 or greater [17]. In light of emerging evidence, the 2023 revision of the FIGO staging system formally incorporated LVSI extent into the staging algorithm, adopting the WHO definition that classifies involvement of ≥5 vessels on a single histologic section as substantial LVSI [14]. A recent study by Dagher et al. [13] found no prognostic distinction between substantial and focal lymphovascular invasion, even among patients with myoinvasive grade 1 or 2 endometrioid endometrial cancer. Notably, focal lymphovascular invasion—defined as involvement of four or fewer vessels on at least one pathology slide—was shown to have a different prognostic impact compared to cases with no lymphovascular invasion [13]. Given the retrospective nature of our study and the inclusion of a substantial number of cases from earlier years, lymphovascular space invasion (LVSI) was evaluated using a binary classification system, recorded simply as either present or absent, in accordance with the pathological reports available at the time.

The prognostic significance of lymphovascular space invasion (LVSI) in early-stage endometrioid endometrial cancer is well established in the existing literature. LVSI positivity rates have been reported in the literature, ranging from 8.3% to 32.8% [15,16,17,18]. The wide variation in these rates may be attributable to different study designs, LVSI definitions, and heterogeneous patient groups in terms of stage and tumor grade. One of the most comprehensive studies on this topic, the LySEC study, evaluated 3308 patients with FIGO 2009 stage I disease and reported a 13.4% prevalence of LVSI. The LVSI positivity rate in our cohort was 17.7%, which is largely consistent with the findings of the LySEC study [19]. In our study, patients without lymphovascular space invasion demonstrated a 5-year disease-free survival rate of 96.3%, compared to 86.4% in those with LVSI. Likewise, the 5-year overall survival rate was 91.2% in the LVSI-negative group and 72.1% in the LVSI-positive group. These survival differences were statistically significant (Log-rank *p* = 0.0020 for DFS; *p* = 0.0014 for OS). Furthermore, univariate Cox regression analysis indicated that LVSI presence was associated with an approximately threefold increase in the risk of disease recurrence (HR = 2.86; 95% CI: 1.43–5.73) and a nearly twofold increase in the risk of overall mortality (HR = 1.95; 95% CI: 1.28–2.97). These outcomes align with those reported by Oliver-Perez et al. in the multicenter LySEC study, which analyzed early-stage endometrioid endometrial cancer patients [19]. In their cohort, 5-year DFS rates were 91.9% for patients without LVSI and 78.9% for those with LVSI, while OS rates were 92.1% and 79.0%, respectively (both *p* < 0.001). Multivariate analysis in that study confirmed LVSI as an independent prognostic indicator for both DFS (HR = 1.9; 95% CI: 1.3–2.5) and OS (HR = 2.1; 95% CI: 1.5–2.9). Collectively, these findings underscore the significance of LVSI as a robust and independent predictor of adverse outcomes in early-stage endometrial cancer, particularly with respect to recurrence and survival [20].

The presence of LVSI consistently emerged as the most significant independent prognostic factor for both OS and DFS. In multivariate analysis, LVSI was associated with a 3.33-fold increased risk of mortality (*p* = 0.012) and a 4.80-fold higher risk of disease recurrence (*p* < 0.001). These findings align with prior studies, including pooled data from the PORTEC trials, where LVSI—particularly in its substantial form—was strongly linked to nodal recurrence, distant metastasis, and decreased survival [16].

In our study, patients with LVSI had larger tumor sizes, higher histologic grades, and deeper myometrial invasion. This supports the notion that LVSI is closely linked to more aggressive tumor biology. Notably, the proportion of grade 3 tumors was higher in the LVSI-positive group (20.5%) compared to the LVSI-negative group (12.4%). Similarly, deep myometrial invasion (≥50%) was markedly more common in the LVSI-positive group (49.4%), reinforcing the association between LVSI and poor prognostic indicators. These results are in line with prior literature and further confirm LVSI as a marker of adverse pathological features [12,19,20].

Interestingly, deep myometrial invasion (>50%) was an independent predictor of reduced DFS (HR: 2.82; *p* = 0.023) but did not significantly affect OS in the multivariate model. This suggests that while deep invasion increases the likelihood of recurrence, it may not necessarily translate into shorter overall survival if recurrences are detected early and treated appropriately. These findings reinforce the importance of myometrial invasion depth in risk stratification models, as reflected in both the ESGO/ESTRO/ESP guidelines and FIGO staging systems [3,4].

In the study by Oliver-Perez et al., recurrence was observed in 278 patients (7.8%) [19]. Of these patients, 17.8% were in the positive LVSI group, and 6.1% were in the negative LVSI group (*p* < 0.001). Regarding the location of recurrence, recurrences limited to the vaginal vault and pelvis were more common in patients with negative LVSI (38.2% vs. 21.7%), while distant recurrences were more common in patients with positive LVSI (61.3% vs. 78.2%) (*p* = 0.01) [19]. Similarly, in our study, recurrence was observed in 37 patients (7.9%) during the follow-up period. The recurrence rate was significantly higher in patients with positive LVSI (14.5% vs. 6.5%, *p* = 0.026). Distant recurrences were predominantly observed in the LVSI-positive group (83.3%), while locoregional recurrences (52.0%) were more common in LVSI-negative patients and were statistically significant (*p* = 0.037). Particularly, distant recurrence was also a major determinant of poor prognosis in our cohort. In multivariate analysis, recurrence was associated with a 4.84-fold increased risk of death (*p* < 0.001) and a 6.12-fold increased risk of disease progression (*p* < 0.001). Distant recurrence independently predicted both OS (HR: 2.52; *p* = 0.032) and DFS (HR: 1.53; *p* < 0.001), reflecting its critical role in long-term disease control. These data corroborate findings from prior studies showing that distant metastatic spread remains the predominant cause of mortality even in early-stage disease [18,19,20,21].

Patients are stratified into risk groups based on stage, histologic grade, and LVSI status, and adjuvant treatment planning is performed accordingly. In the low-risk group, adjuvant therapy is not recommended. In the intermediate-risk group, adjuvant brachytherapy may be considered to reduce the risk of vaginal recurrence; in particular, omission of adjuvant brachytherapy may be considered in patients younger than 60 years. The high–intermediate-risk group includes patients with substantial LVSI and those with stage IB, grade 3 tumors regardless of LVSI status. For these patients, adjuvant brachytherapy may be recommended to reduce the risk of vaginal recurrence, and adjuvant chemotherapy may be considered, especially in cases with high-grade tumors and/or substantial LVSI [3]. In our study, adjuvant treatment patterns also varied significantly based on LVSI status. More than two-thirds of LVSI-positive patients received external beam radiotherapy, compared to a significantly lower proportion in the LVSI-negative group. Adjuvant treatment was independently associated with poorer DFS (HR: 4.32; *p* = 0.044) and OS (HR: 5.45; *p* = 0.047) in multivariate analyses. This seemingly paradoxical finding is likely attributable to treatment indication bias, whereby patients with high-risk features (e.g., LVSI, high grade, deep invasion) are more likely to receive adjuvant therapy. This indicates that clinicians often incorporate LVSI status into postoperative treatment planning, highlighting its real-world relevance in guiding therapeutic decisions.

Additionally, in patients with endometrial cancer who are candidates for fertility-sparing treatment, preserving fertility prior to oncologic management is of paramount importance. Fertility preservation strategies such as oocyte vitrification have been shown to enhance the likelihood of achieving pregnancy following treatment and provide patients with the opportunity to maintain their reproductive potential [22,23]. Furthermore, the neonatal outcomes and long-term follow-up of children born from frozen embryos represent critical considerations for ensuring the safety of fertility-sparing approaches. Recent studies have highlighted the need for close monitoring of neurodevelopmental and cardiovascular outcomes in these children. Comparative studies on fresh versus frozen embryo transfer have provided valuable insights into the clinical outcomes and ethical–legal implications of these assisted reproductive techniques [24,25]. In light of current evidence, fertility preservation strategies should be planned in a multidisciplinary manner, considering not only oncologic safety but also obstetric and neonatal outcomes, as well as the medico-legal context [23,25].

### Strengths and Limitations

The main strengths of our study are the inclusion of a large patient cohort supported by long-term follow-up data and the homogeneous nature of the cohort. By excluding patients with non-endometrioid histologies and nodular involvement, we were able to more accurately assess the independent prognostic role of LVSI. Furthermore, consistent surgical and pathological evaluation protocols at a single center increased data reliability.

However, the retrospective design of the study introduces a potential methodological limitation in the form of selection bias, albeit to a limited extent. Additionally, the extent of LVSI (focal vs. substantial) was not assessed. Another notable limitation is the absence of molecular profiling data. Nevertheless, considering that the 2023 FIGO classification accounts for clinical scenarios in which molecular testing is not available, our study offers valuable insights by reflecting real-world clinical practice.

During the extended study period, particularly in the earlier years of the cohort, sentinel lymph node (SLN) mapping was not implemented as a standard staging procedure. SLN biopsy only gained acceptance and began to be utilized following the publication of robust scientific evidence in recent years demonstrating its efficacy and reliability [26]. Although our institution is a tertiary referral center, the lack of a near-infrared imaging system currently precludes SLN mapping with indocyanine green (ICG). In selected cases, SLN biopsy was performed using methylene blue; however, due to a low detection success rate, this approach was applied in only a limited number of patients. The fact that approximately 80% of patients underwent laparotomy similarly reflects the surgical practice patterns of the earlier years of the study period. At that time, access to minimally invasive surgical techniques was limited, and the widespread adoption of laparoscopic surgery occurred only in recent years, in parallel with improvements in technological capabilities, increased surgical experience, and changes in international guidelines.

## 5. Conclusions

This study demonstrates that the presence of lymphovascular space invasion is a strong and independent prognostic factor for both disease-free survival and overall survival in patients with stage I endometrioid endometrial cancer with negative lymph nodes. LVSI was significantly associated with unfavorable pathological features, higher recurrence rates, and increased cancer-related mortality. Multivariate analysis confirmed that LVSI independently increased the risk of recurrence nearly fivefold and the risk of death more than threefold. Distant recurrence was more common in LVSI-positive patients and emerged as a major determinant of poor prognosis. These findings are in concordance with previous large-scale studies and further validate the inclusion of LVSI in contemporary staging and risk stratification systems. Our results also highlight the clinical importance of incorporating LVSI status into adjuvant treatment decision-making. Future prospective studies with molecular profiling may provide additional refinement in identifying patients who would most benefit from intensified surveillance or therapy.

## Figures and Tables

**Table 1:** Demographic, clinical, and pathologic characteristics of patients with stage I endometrioid endometrial cancer according to lymphovascular space invasion.

Characteristics	Total (*n* = 469)	LVSI Positive *n*: 83 (17.7%)	LVSI Negative *n*: 386 (82.3%)	*p*-Value
Age, y, median (range)	61.4 (27–90)	63.0 (39–90)	61.0 (27–85)	0.094
BMI, kg/m^2^, median (range)	34.7 (18.0–65.35)	35.3 (22.06–65.35)	34.6 (18.0–64.0)	0.708
Menopause status	431 (91.9%)	78 (94.0%)	353 (91.5%)	0.587
Tumor size (cm), median (range)	3.5 (0.2–11.0)	4.5 (1.0–10.0)	3.3 (0.2–11.0)	**<0.001**
FIGO grade, *n* (%)				**<0.001**
G1	256 (54.6%)	26 (31.3%)	230 (59.6%)	
G2	148 (31.6%)	40 (48.2%)	108 (28.0%)	
G3	65 (13.9%)	17 (20.5%)	48 (12.4%)	
Depth of myometrial invasion, *n* (%)				**<0.001**
None	31 (6.6%)	1 (1.2%)	30 (7.8%)	
<50%	311 (66.3%)	41 (49.4%)	270 (69.9%)	
≥50%	127 (27.1%)	41 (49.4%)	86 (22.3%)	
FIGO 2009 stage, (*n*, %)				**<0.001**
1A	341 (72.7%)	43 (51.8%)	298 (77.2%)	
1B	128 (27.3%)	40 (48.2%)	88 (22.8%)	
Risk groups				**<0.001**
Low risk	263 (56.1%)	0 (0.0%)	263 (68.1%)	
Intermediate risk	110 (23.5%)	0 (0.0%)	110 (25.6%)	
High-Intermediate risk	96 (20.5%)	83 (100%)	13 (3.4%)	
Surgical approach, (*n*, %)	110 (23.5%)			0.925
Laparoscopy		16 (19.3%)	79 (20.5%)	
Laparotomy		67 (80.7%)	307 (79.5%)	
Adjuvant therapy, (*n*, %)	96 (20.5%)			**<0.001**
No adjuvant therapy		3 (3.6%)	166 (43.0%)	
Chemotherapy		8 (9.6%)	10 (2.6%)	
EBRT		55 (66.3%)	147 (38.1%)	
Brachytherapy		17 (20.5%)	63 (16.3%)	
Para-aortic LN dissection (*n*, %)	358 (76.3%)	70 (84.3%)	288 (74.6%)	0.080
No. of PALN harvested, median (range)	10.8 (1–46)	13.5 (1–41)	10.0 (1–46)	0.053
No. of PLN harvested, median (range)	19.1 (2–82)	20.0 (2–49)	19.0 (2–82)	0.620
Positive peritoneal cytology, (*n*, %)	5 (1.1%)	2 (2.4%)	3 (0.8%)	0.469
Recurrence, *n* (%)				**0.026**
Yes	37 (7.9%)	12 (14.5%)	25 (6.5%)	
No	432 (92.1%)	71 (85.5%)	361 (93.5%)	
Recurrence site				**0.037**
Locoregional	14 (37.8%)	1 (8.3%)	13 (52.0%)	
Distant	21 (56.8%)	10 (83.3%)	11 (44.0%)	
Locoregional + Distant	2 (5.4%)	1 (8.3%)	1 (4.0%)	
Death, *n* (%)	126 (26.9%)	29 (34.9%)	97 (25.1%)	0.091
Cause of death, *n* (%)				0.495
Cancer-related	35 (7.5%)	10 (34.5%)	25 (25.8%)	
All other causes	91 (19.4%)	19 (65.5%)	72 (74.2%)	

PALN; para-aortic lymph node, PLN; pelvic lymph node, y; years. Bold values indicate statistical significance.

**Table 2:** Univariate and multivariate analysis of factors affecting overall survival.

	OS			
Characteristics	Univariate		Multivariate	
	HR (95% CI)	*p*-Value	HR (95% CI)	*p*-Value
LVSI Positive	3.91 (1.92–8.01)	**<0.001**	3.33 (1.43–7.77)	**0.012**
Age ≥ 60 y.	1.83 (1.23–2.62)	**0.005**	1.02 (0.97–1.07)	0.245
BMI	0.97 (0.92–1.03)	0.292	0.99 (0.95–1.04)	0.612
Menopause status	2.02 (0.94–4.35)	0.084	1.79 (0.37–8.72)	0.473
Grade 3	2.51 (1.13–5.57)	**0.041**	1.13 (0.40–3.20)	0.822
Tumor size ≥ 2 cm	2.03 (1.12–3.74)	**0.023**	1.03 (0.82–1.30)	0.791
MI ≥ 50%	1.81 (0.93–3.54)	0.094	2.13 (0.81–2.64)	0.186
Para-aortic LN dissection (yes)	1.66 (0.86–3.17)	0.133	2.00 (0.53–7.52)	0.312
Surgical approach (Laparotomy)	0.68 (0.30–1.26)	0.141	0.23 (0.04–1.27)	0.092
FIGO 2009 stage IB	2.24 (1.14–5.12)	**0.049**	2.31 (0.24–22.53)	0.473
Adjuvant treatment (received)	1.54 (0.73–3.38)	0.223	5.45 (1.02–29.10)	**0.047**
Recurrence (yes)	5.73 (2.91–11.33)	**<0.001**	4.84 (1.94–11.84)	**<0.001**
Recurrence site (distant)	3.29 (1.55–6.82)	**0.002**	2.52 (1.12–5.73)	**0.032**

MI ≥ 50%; myometrial invasion equal to or greater than 50%, y; years. Bold values indicate statistical significance.

**Table 3:** Univariate and multivariate analysis of factors affecting disease-free survival.

	DFS			
Characteristics	Univariate		Multivariate	
	HR (95% CI)	*p*-Value	HR (95% CI)	*p*-Value
LVSI Positive	4.21 (2.02–8.61)	**<0.001**	4.80 (1.62–14.21)	**<0.001**
Age ≥ 60 y.	1.63 (0.93–3.05)	0.122	1.03 (0.98–1.09)	0.243
BMI	0.95 (0.89–1.02)	0.153	0.96 (0.92–1.12)	0.534
Menopause status	1.84 (0.84–3.93)	0.136	1.71 (0.45–5.73)	0.623
Grade 3	2.05(1.06–4.35)	**0.031**	1.24 (0.46–2.28)	0.324
Tumor size ≥ 2 cm	2.23 (1.14–4.49)	**0.037**	1.16 (0.64–1.36)	0.245
MI ≥ 50%	2.37 (1.01–5.38)	**0.045**	2.82 (1.24–2.93)	**0.023**
Para-aortic LN dissection (yes)	1.54 (0.74–3.13)	0.236	1.70 (0.66–5.56)	0.634
Surgical approach (Laparatomy)	0.76 (0.42–1.45)	0.294	0.42 (0.13–1.29)	0.076
FIGO 2009 stage IB	2.17 (1.08–4.35)	**0.041**	2.12 (0.32–12.43)	0.345
Adjuvant treatment (received)	1.18 (0.83–3.93)	0.191	4.32 (1.02–26.13)	**0.044**
Recurrence (yes)	6.21 (3.82–10.71)	**<0.001**	6.12 (4.93–9.77)	**<0.001**
Recurrence site (distant)	2.45 (1.12–4.65)	**<0.001**	1.53 (1.34–6.54)	**<0.001**

MI ≥ 50%; myometrial invasion equal to or greater than 50%, y; years. Bold values indicate statistical significance.

## Data Availability

The original contributions presented in this study are included in the article. Further inquiries can be directed to the corresponding author(s).

## Abbreviations

The following abbreviations are used in this manuscript:

LVSI	Lymphovascular space invasion
DFS	Disease-free survival
OS	Overall survival
EEC	Endometrioid endometrial cancer
FIGO	International Federation of Obstetrics and Gynecology
LN	Lymph node
NCCN	National Comprehensive Cancer Network
GOG-99	Gynecologic Oncology Group Study 99
ESGO	European Society of Gynaecological Oncology
WHO	World Health Organization
BMI	Body Mass Index
EBRT	External Beam Radiotherapy
MI	Myometrial Invasion
ESTRO	European Society for Radiotherapy and Oncology
ESP	European Society of Pathology
